# Hemodynamic effects of intraoperative 30% versus 80% oxygen concentrations: an exploratory analysis

**DOI:** 10.3389/fmed.2023.1200223

**Published:** 2023-05-30

**Authors:** Christian Reiterer, Edith Fleischmann, Barbara Kabon, Alexander Taschner, Andrea Kurz, Nikolas Adamowitsch, Markus Falkner von Sonnenburg, Melanie Fraunschiel, Alexandra Graf

**Affiliations:** ^1^Department of Anesthesia, Intensive Care Medicine and Pain Medicine, Medical University of Vienna, Vienna, Austria; ^2^Outcome Research Consortium, Cleveland, OH, United States; ^3^Department of General Anesthesiology, Cleveland Clinic, Anesthesia Institute, Cleveland, OH, United States; ^4^Department of General Anesthesiology, Emergency Medicine and Intensive Care Medicine, Medical University of Graz, Graz, Austria; ^5^IT Systems and Communications, Medical University of Vienna, Vienna, Austria; ^6^Center for Medical Data Science, Medical University of Vienna, Vienna, Austria

**Keywords:** supplemental oxygen, hemodynamics, blood pressure, heart rate, major abdominal surgeries

## Abstract

**Background:**

Supplemental oxygen leads to an increase in peripheral vascular resistance which finally increases systemic blood pressure in healthy subjects and patients with coronary artery disease, heart failure, undergoing heart surgery, and with sepsis. However, it is unknown whether this effect can also be observed in anesthetized patients having surgery. Thus, we evaluated in this exploratory analysis of a randomized controlled trial the effect of 80% versus 30% oxygen on intraoperative blood pressure and heart rate.

**Methods:**

We present data from a previous study including 258 patients, who were randomized to a perioperative inspiratory FiO_2_ of 0.8 (128 patients) versus 0.3 (130 patients) for major abdominal surgery. Continuous arterial blood pressure values were recorded every three seconds and were exported from the electronic anesthesia record system. We calculated time-weighted average (TWA) and Average Real Variability (ARV) of mean arterial blood pressure and of heart rate.

**Results:**

There was no significant difference in TWA of mean arterial pressure between the 80% (80 mmHg [76, 85]) and 30% (81 mmHg [77, 86]) oxygen group (effect estimate −0.16 mmHg, CI –1.83 to 1.51; *p* = 0.85). There was also no significant difference in TWA of heart rate between the 80 and 30% oxygen group (median TWA of heart rate in the 80% oxygen group: 65 beats.min^−1^ [58, 72], and in the 30% oxygen group: 64 beats.min^−1^ [58; 70]; effect estimate: 0.12 beats.min^−^1, CI –2.55 to 2.8, *p* = 0.94). Also for ARV values, no significant differences between groups could be detected.

**Conclusion:**

In contrast to previous results, we did not observe a significant increase in blood pressure or a significant decrease in heart rate in patients, who received 80% oxygen as compared to patients, who received 30% oxygen during surgery and for the first two postoperative hours. Thus, hemodynamic effects of supplemental oxygen might play a negligible role in anesthetized patients.

**Clinical Trail Registration:**

https://clinicaltrials.gov/ct2/show/NCT03366857?term=vienna&cond=oxygen&draw=2&rank=1

## Highlights

–Question: Has the administration of supplemental oxygen a significant effect on blood pressure and heart rate?

–Findings: We did not find a significant difference in blood pressure and heart between patients receiving 80% versus 30% oxygen during major abdominal surgery.

–Meaning: The administration of supplemental oxygen has no significant effect on intraoperative blood pressure and heart rate in patients undergoing major abdominal surgery.

## Background

The administration of oxygen counts to one of the most common treatments of hospitalized patients. These include patients on the intensive care unit, perioperative patients, emergency patients and patients on the ward (1–5). Former studies showed that the administration of supplemental oxygen leads to increased postoperative mortality, larger infarct size in patients with ST-segment elevation myocardial infarction, increased cerebral infarct size, and higher mortality in intensive care patients (1, 4, 6). However, our main trial and also other trials did not detect any harmful effects when 80% of oxygen was given, specifically in the perioperative period (5, 7–9). In fact, recent follow-up studies show that higher oxygen concentrations were not associated with higher mortality (10, 11).

Although supplemental oxygen has no impact on mortality, myocardial ischemia, pulmonary atelectasis, oxidative stress, several studies described a significant hemodynamic effect when 80% oxygen was administered (12). Some smaller studies and one recent meta-analysis showed that oxygen leads to an increase in blood pressure, a reduction in cardiac output, and a decrease in heart rate in healthy volunteers, patients with coronary heart disease, heart failure, CABG, and septic patients (12). One possible explanation for these hemodynamic effects is the fact that an increase in oxygen concentration leads to a significant increase of peripheral vascular resistance in isolated skeletal muscle arterioles (13). In this context the authors observed that short-term hyperoxia results in an inhibition of endothelial prostaglandin synthesis leading to vasoconstriction. In addition, decrease in heartrate might further be explained by the finding that the administration of oxygen leads to a dose-dependent increase in parasympathetic activity and arterial-cardiac baroreflex function (14).

In the perioperative setting, the concentration of administered oxygen varies significantly and is mainly based on individual preferences of the attending anesthesiologists. While the effects of perioperative oxygen administration on postoperative outcomes such as wound infections, long-term mortality or myocardial injury have been investigated intensively, the detailed effects on intraoperative hemodynamic parameters have not been evaluated before (2, 3, 5, 7, 10, 11, 15, 16).

Based on previous studies, we hypothesized that the administration of 80% oxygen leads to an increase in TWA of intraoperative blood pressure and a reduction in TWA of intraoperative heart rate in patients at-risk for cardiovascular complications undergoing moderate- to high-risk major noncardiac surgery. We further analyzed the effect of 80% versus 30% oxygen on intraoperative average real variability (ARV).

## Methods

This study was approved by the University’s Institutional Review Board (EK: 1477/2017) and written informed consent was obtained from all subjects participating in the trial. The trial was registered prior to patient enrollment at ClinicalTrials.gov (NCT03366857, Principal investigator: Edith Fleischmann, https://clinicaltrials.gov/ct2/show/NCT03366857?cond=NCT03366857&draw=2&rank=1; Date of Registration: 8th December 2017) and was further registered at the and the European Trial Database (EudraCT 2017–003714-68).

We conducted exploratory analyses of the observed data on intraoperative mean arterial blood pressure and heart rate of our randomized trial in which we investigated the effect of 80% versus 30% oxygen on postoperative maximum NT-proBNP concentration as primary hypothesis in 260 patients at-risk for cardiovascular complications undergoing moderate- to high-risk abdominal surgery (7). The study was performed at the Medical University of Vienna. We conducted the trial according to the Declaration of Helsinki and Good Clinical Practice. The results of the primary outcome and several sub-analyses were published previously (7, 17–19).

Patients, who were at least 45 years of age and had at least one cardiovascular risk factor have been included in our original trial. Detailed inclusion and exclusion criteria and a detailed description of patient characteristics have been published previously (7). Patients, who met the following criteria have been included: moderate- to high-risk abdominal surgery expected to last ≥ two hours, at least 45 years, and one of the following criteria (1) history of coronary artery disease, (2) history peripheral artery disease, (3) history of stroke, (4) or any three of the following criteria: (a) age over 70 years, (b) undergoing major surgery, (c) diabetes and currently taking an oral hypoglycemic agent or insulin, or (d) history of hypertension. Our exclusion criteria were: (1) sepsis, (2) preoperative inotropic therapy, (3) oxygen dependence, or (4) history of severe heart failure (left ventricular ejection fraction lower than 30%).

We randomly assigned patients to receive 80% versus 30% oxygen concentration throughout surgery and for the first two postoperative hours. We included 260 patients between December 2017 and December 2019. Patients received the assigned oxygen concentration after endotracheal intubation. Patients, who were assigned to the 80% oxygen group received 8 L.min^−1^ *via* facemask with reservoir, and patients, who were assigned to the 30% oxygen group received 3 L.min^−1^ for the first two postoperative hours.

We performed esophagus guided fluid management in all patients according to a previously published algorithm. We aimed to held blood pressure within 20% of preoperative baseline values. All patients received an arterial line for continuous blood pressure measurement. From the underlying trial we extracted arterial blood pressure values with a 1-min resolution. Other than oxygen concentration, anesthetic management was at the discretion of the anesthesia team.

### Statistical analysis

We summarized continuous variables using mean, standard deviation, median, quartiles as well as minimum and maximum. Descriptive statistics are given for both randomization groups separately.

To investigate the influence of the group assignment on the time weighted average (TWA), average real variability (ARV), generalized ARV as well as squared ARV (all measured in mmHg/min) of mean, diastolic and systolic arterial blood pressure as well as heart frequency, we first calculated univariable linear regression models. Hansen et al. (20) proposed the ARV [see formula ARV (1)] as a parameter to estimate the variability for time-series data. However, the proposed formula may overestimate the variability of steep changes for blood pressure measurements with non-equal time intervals. Mascha et al. therefore proposed the generalized ARV [see generalized ARV formula (2)], which is the sum of absolute value of all changes of all measurements divided by total time (21). Equal time measurements are not required for the calculation of the generalized ARV (21). Furthermore, the authors proposed a squared version of the generalized ARV which gives more weight to steep changes of the blood pressure [see formula squared ARV (3)]. We calculated ARV (1), generalized ARV (2), and squared ARV (3) according to Mascha et al. (21):


(1) $$\mathrm{ARV}=\frac{1}{\sum t}\overset{N=1}{\underset{k=1}{\sum }}t\;\left|{\mathrm{BP}}_{k+1}-{\mathrm{BP}}_{k}\right|\mathrm{mmHg}/\min,$$

(2) $$\mathrm{Generalized}\;\mathrm{ARV}=\frac{1}{T}\overset{N=1}{\underset{k=1}{\sum }}\;\left|{\mathrm{BP}}_{k+1}-BP_{k}\right|\mathrm{mmHg}/\min,$$

(3) $$\mathrm{Squared}\;\mathrm{ARV}=\frac{\frac{1}{\sum t}\sum_{k=1}^{N=1}\frac{\;{\left|{\mathrm{BP}}_{k+1}-{\mathrm{BP}}_{k}\right|}^{2}}{t_{k+1}-t_{k}}\mathrm{mmHg}}{\min}.$$

Additionally, potential confounding factors as age, BMI, sex (male vs. female), type of surgery (laparoscopic vs. open), history of coronary artery disease, peripheral artery disease, stroke, heart failure, diabetes as well as hypertension, vasopressor use, Albumin administration (yes vs. no), crystalloids, the time weighted average of pCO_2_, as well as pH, were analyzed using univariable linear regression models. Additionally, if more than one influence parameter showed a significant result in the univariable models (*p* < 0.05), we performed a multivariable linear regression model accounting for all factors being significant in the univariable analyses.

Due to the exploratory character of the study, we did not perform a correction for multiplicity. All *p*-values smaller or equal to 0.05 were considered as statistically significant. Detailed discussion on the calculation of ARV has been published previously (21). All analyses were performed using R, release 4.2.2.

## Results

The underlying trial was conducted at the Medical University of Vienna from 2017 to 2019 and included 258 patients, who had moderate to major abdominal surgery. Two patients were excluded because surgery was postponed after randomization. In the current trial we present the analysis of hemodynamic data (intraoperative arterial blood pressure and heart rate) from 258 patients, who were randomly assigned to 80% versus 30% perioperative inspired oxygen concentration. 127 patients received 80% oxygen and 130 patients received 30% oxygen. Baseline characteristics between the groups were well balanced and published previously (7).

### Time-weighted average (TWA) of mean arterial blood pressure

**Figure 1:**
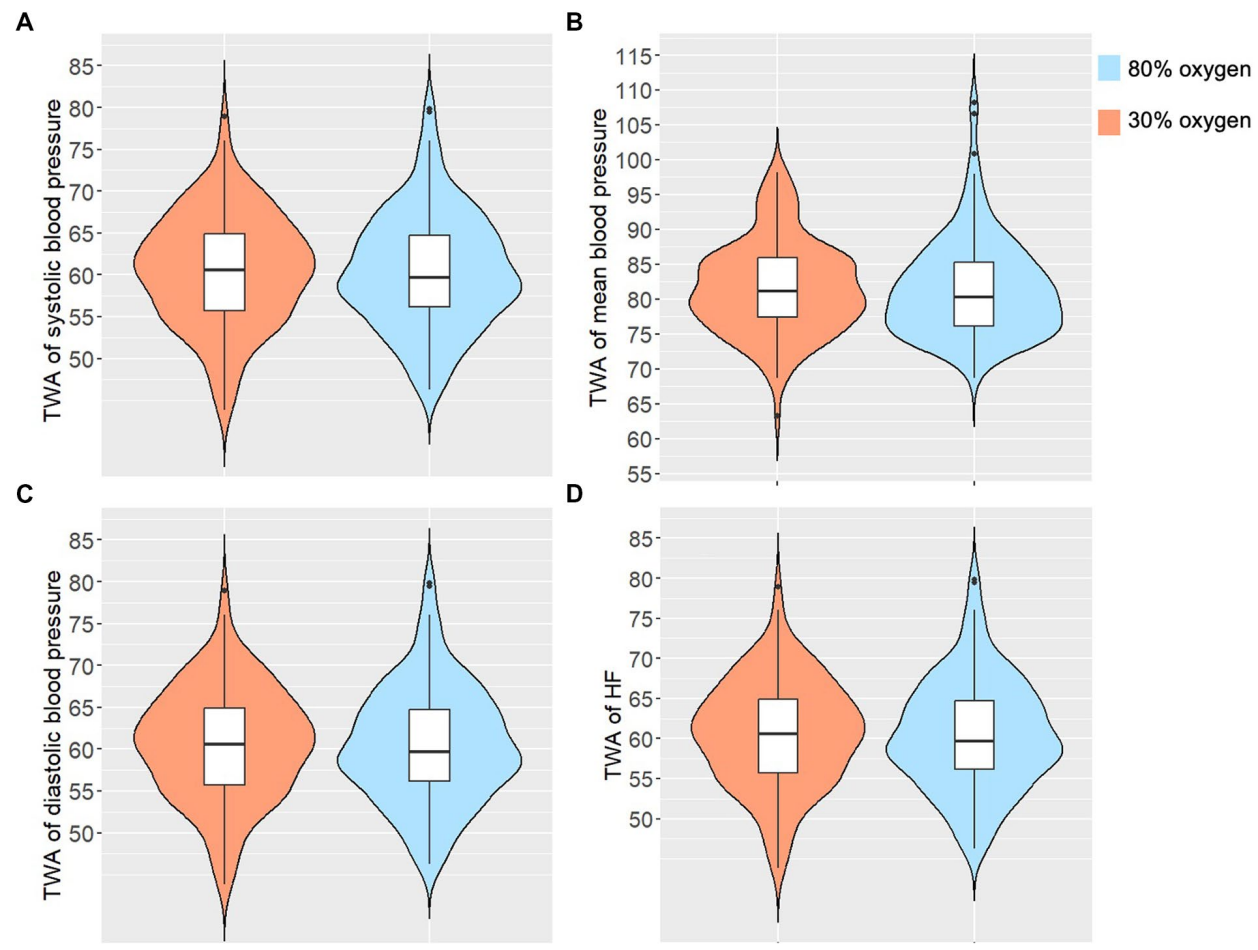
The Volin-plot shows the distribution of **(A)** time-weighted average (TWA) of systolic blood pressure, **(B)** TWA of median blood pressure, **(C)** TWA of diastolic blood pressure, and **(D)** TWA of heart rate (HF) separately for the groups.

We did not observe a significant effect of 80% versus 30% perioperative oxygen on TWA of mean arterial blood pressure (Figure 1). Detailed descriptive analyses of the TWA of mean, diastolic and systolic blood pressure are presented in Table 1.

**Table 1:** Descriptive statistics of TWA, ARV, generalized ARV and squared ARV of systolic blood pressure separately for the randomization groups.

	Variable	Group	Mean	SD	Median	Q1	Q3	*n*
Systolic	TWA	80%	121	11	118	113	127	127
		30%	122	12	121	113	129	130
	ARV	80%	4.5	1.5	4.2	3.6	5.2	127
		30%	4.8	1.8	4.4	3.8	5.2	130
	Generalized ARV	80%	16.0	4.8	15.3	12.7	5.2	127
		30%	16.4	4.3	15.6	13.4	5.2	130
	Squared ARV	80%	1424.5	889.1	1205.5	877.1	1740.3	127
		30%	1573.0	971.2	1366.1	892.2	2022.8	130
Mean	TWA	80%	82	7	80	76	85	127
		30%	82	7	81	77	86	130
	ARV	80%	3.1	1.4	2.8	2.4	3.5	127
		30%	3.1	1.0	2.9	2.6	3.5	130
	Generalized ARV	80%	10.6	3.0	10.0	8.5	12.6	127
		30%	10.8	2.9	10.4	9.0	12.2	130
	Squared ARV	80%	540.0	288.9	472.3	333.8	676.7	127
		30%	594.9	357.4	477.9	362.6	742.5	130
Diastolic	TWA	80%	60.5	6.7	60.6	55.7	64.9	127
		30%	60.6	6.9	59.6	56.2	64.7	130
	ARV	80%	2.8	1.5	2.6	2.0	3.1	127
		30%	2.7	0.9	2.5	2.1	3.0	130
	Generalized ARV	80%	9.1	2.9	8.5	7.2	10.3	127
		30%	9.1	2.4	8.8	7.5	10.4	130
	Squared ARV	80%	459.2	285.2	386.8	285.3	543.0	127
		30%	461.8	266.9	389.7	286.6	579.0	130
Heart rate	TWA	80%	66	11	65	58	72	128
		30%	66	11	64	58	70	130
	ARV	80%	3.2	1.9	2.4	1.9	4.3	128
		30%	3.2	1.9	2.7	1.8	4.3	130
	Generalized ARV	80%	11.9	6.9	8.9	7.1	15.4	128
		30%	11.9	7.0	9.8	6.9	15.6	130
	Squared ARV	80%	1005.1	887.3	724.7	370.7	1262.6	128
		30%	1093.6	938.8	774.6	416.7	1505.7	130

Summary characteristics are presented as mean and standard deviation (SD), and median and quartiles (Q1, 25th percentile; Q3, 75th percentile). TWA, time-weighted average; ARV, average real variability.

Median TWA of mean blood pressure was 80 mmHg [Interquartil Range (IQR): 76; 85] in the 80% oxygen group and 81 mmHg [77, 86] in the 30% oxygen group (effect estimate: −0.16 mmHg, 95%-CI –1.83–1.51; *p* = 0.85). A significantly larger TWA of mean arterial blood pressure was found for laparoscopic surgery as compared to open surgery (effect estimate 2.91 mmHg, CI 1.19–4.62, *p* < 0.01). Furthermore, a significant smaller TWA of MAP was found for patients, who received vasopressors (*p* < 0.01), and patients, who received Albumin (*p* = 0.03). Larger TWA of MAP values were found for decreasing amount of crystalloids (*p* = <0.01) and increasing pH values (*p* < 0.01). In the multivariable analysis accounting for type of surgery, vasopressor use, Albumin administration, amount of crystalloids, and pH, the factors vasopressor use and pH remained significant (Online Supplementary eTable) Detailed results of analyses of TWA of mean arterial pressure are presented in Table 2. Note that also for the TWA of the systolic or diastolic blood pressure, no significant effect of 80% versus 30% perioperative oxygen was observed (Online Supplementary eTables 3, 5).

**Table 2:** Univariable regression models for TWA, ARV, generalized ARV and squared ARV of the intraoperative mean arterial blood pressure.

	Variable	Comparison	Estimate	95% CI	*p*-Value
TWA	Randomization Group	80 vs. 30% oxygen	−0.16	−1.83–1.51	0.85
	Age		0.10	−0.01–0.21	0.08
	BMI		0.02	−0.15–0.19	0.84
	Sex	Male vs. Female	1.47	−0.30–3.23	0.10
	Type of surgery	Laparoscopic vs. Open	2.91	1.20–4.62	< 0.01
	Coronary artery disease	Yes vs. no	0.26	−1.68–2.20	0.79
	Peripheral artery disease	Yes vs. no	−0.69	−3.01–1.64	0.56
	Stroke	Yes vs. no	0.41	−2.57–3.40	0.79
	Heart failure	Yes vs. no	1.04	−2.23–4.31	0.53
	Diabetes	Yes vs. no	−1.18	−3.05– 0.68	0.21
	Hypertension	Yes vs. no	2.58	−0.67 – 5.84	0.12
	Vasopressor	Yes vs. No	−4.90	−7.08 – −2.72	< 0.01
	Albumin	Yes vs. No	−1.88	−3.61 – −0.15	0.03
	Crystalloids		−0.98	−1.53 – −0.44	<0.01
	TWA: pCO_2_		0.02	−0.17 – 0.20	0.87
	TWA: pH		24.14	6.09–42.19	<0.01
ARV	Randomization Group	80% vs. 30% oxygen	−0.01	−0.31 – 0.3	0.93
	Age		0.03	0.01–0.05	<0.01
	BMI		−0.03	−0.06 – 0.01	0.10
	Sex	Male vs. Female	−0.25	−0.56 – 0.07	0.12
	Type of surgery	Laparoscopic vs. Open	0.20	−0.11 – 0.51	0.20
	Coronary artery disease	Yes vs. No	−0.09	−0.43 – 0.25	0.60
	Peripheral artery disease	Yes vs. No	0.19	−0.22 – 0.60	0.36
	Stroke	Yes vs. No	−0.18	−0.71 – 0.34	0.49
	Heart failure	Yes vs. No	0.19	−0.39 – 0.76	0.52
	Diabetes	Yes vs. No	−0.31	−0.63 – 0.02	0.07
	Hypertension	Yes vs. No	−0.17	−0.75 – 0.40	0.56
	Vasopressor	Yes vs. No	−1.10	−0.50 – −2.29	0.61
	Albumin	Yes vs. No	−0.02	−0.28 – −0.33	0.88
	Crystalloids		−0.16	−0.25 – −0.06	0.01
	TWA: pCO_2_		0.02	−0.01 – 0.06	0.13
	TWA: pH		−2.53	−5.73 – 0.66	0.12
Generalized ARV	Randomization Group	80 vs. 30% oxygen	−0.25	−1.00 – 0.47	0.50
	Age		0.11	0.06–0.15	< 0.01
	BMI		−0.12	−0.19 – −0.05	< 0.01
	Sex	Male vs. Female	−0.81	−1.57 – −0.04	0.04
	Type of surgery	Laparoscopic vs. Open	0.01	−0.75 – 0.77	0.980
	Coronary artery disease	Yes vs. No	−0.48	−1.33 – 0.36	0.26
	Peripheral artery disease	Yes vs. No	−0.02	−1.03 – 1.00	0.98
	Stroke	Yes vs. No	−0.52	−1.82 – 0.77	0.43
	Heart failure	Yes vs. No	−0.50	−1.92 – 0.92	0.49
	Diabetes	Yes vs. No	−0.87	−1.68 – −0.07	0.04
	Hypertension	Yes vs. No	−0.51	−1.93 – 0.91	0.49
	Vasopressor	Yes vs. No	0.84	−1.14 – 1.82	0.09
	Albumin	Yes vs. No	0.08	−0.67 – 0.84	0.92
	Crystalloids		−0.37	−0.61 – −0.13	<0.01
	TWA: pCO_2_		−0.03	−0.11 – 0.06	0.52
	TWA: pH		−1.76	−9.71 – 6.19	0.66
Squared ARV	Randomization Group	80 vs. 30% oxygen	−54.95	−134.51 – 24.6	0.18
	Age		4.48	−0.76 – 9.7	0.10
	BMI		−8.24	−16.36 – −0.1	0.05
	Sex	Male vs. Female	1.13	−83.72 – 86.0	0.98
	Type of surgery	Laparoscopic vs. Open	81.57	−1.51 – 164.7	0.06
	Coronary artery disease	Yes vs. No	−14.59	−107.37 – 78.2	0.76
	Peripheral artery disease	Yes vs. No	−2.42	−113.68 – 108.84	0.97
	Stroke	Yes vs. No	−15.76	−158.42 – 126.91	0.83
	Heart failure	Yes vs. No	−74.58	−230.72 – 81.57	0.35
	Diabetes	Yes vs. No	−33.10	−122.29 – 56.08	0.47
	Hypertension	Yes vs. No	−97.29	−253.25 – 58.66	0.22
	Vasopressor	Yes vs. No	16.73	−91.21 – 124.68	0.76
	Albumin	Yes vs. No	−23.41	−106.63 – 59.81	0.58
	Crystalloids		−51.45	−77.26 – −25.64	<0.01
	TWA: pCO_2_		0.33	−8.64 – 9.29	0.94
	TWA: pH		−245.33	−1119.62 – 628.96	0.58

Estimate of slope, confidence intervals (CI) and p-values were calculated using univariable linear regression models. TWA, time-weighted average; ARV, average real variability.

### Average real variability, generalized ARV, and squared ARV of mean arterial blood pressure

We did not observe a significant difference between 80 and 30% oxygen in ARV (*p* = 0.93), generalized ARV (*p* = 0.50) or squared ARV (*p* = 0.18) of mean arterial blood pressure. Detailed descriptive analysis of mean, diastolic and systolic blood pressure are presented in Table 1.

Median ARV of mean arterial blood pressure was 2.8 mmHg [2.4; 3.5] in the 80% oxygen group and 2.9 mmHg [2.6, 3.5] in the 30% oxygen group (effect estimate −0.01 mmHg, CI –0.31–0.28; *p* = 0.93).

A significant larger ARV of mean arterial blood pressure was found for older patients (*p* < 0.01). A significant larger ARV of the MAP was found for older patients (*p* < 0.01). Larger ARV values were found for decreased amount of crystalloids (*p* < 0.01; Table 2). In the multivariable analysis accounting for age and crystalloids, both factors remained significant (Online Supplementary eTable 2).

We observed a significant larger generalized ARV of mean arterial pressure for older patients (*p* < 0.01), smaller BMI (*p* < 0.01), females (*p* = 0.01) and patients without diabetes (*p* = 0.04). Larger generalized ARV values were found for decreasing amount of crystalloids (*p* < 0.01; Table 2). Age, BMI, and amount of crystalloids remained significant in the multivariable analysis (age: estimate 0.08 mmHg, CI 0.02–0.13, *p* < 0.01; BMI: estimate: −0.10 mmHg, CI –0.17 to −0.02, *p* = 0.01; gender: estimate: –0.75, CI –1.48 to −0.01, *p* = 0.05; diabetes: estimate: −0.39 mmHg, CI –1.20 to 0.42, *p* = 0.35; Online Supplementary eTable 2).

A significant smaller squared ARV of mean arterial blood pressure was found for patients with larger BMI (*p* = 0.05). Larger squared ARV values were found for decreasing amount of crystalloids (*p* < 0.01; Table 2). In the multivariable analysis accounting for BMI and amount of crystalloids, only the factor crystalloids remained significant (Online Supplementary eTable 2).

Note that also for the ARV, generalized ARV and squared ARV of the systolic or diastolic blood pressure, no significant effect of 80% versus 30% perioperative oxygen was observed (Online Supplementary eTables 3–6).

### Heart rate

No significant difference in TWA of heart rate between patients receiving 80% versus 30% oxygen was observed (median TWA of heart rate in the 80% oxygen group: 65 beats.min^−1^ [58; 72], and in the 30% oxygen group: 64 beats.min^−1^ [58; 70]; effect estimate: 0.12 beats.min^−^1, CI -2.55 to 2.80, *p* = 0.94).

Time-weighted average of heart rate was significant higher in patients with a lower BMI (*p* = 0.04), patients, who had an open surgery (*p* < 0.01) as well as in patient without coronary artery diseases (*p* = 0.03). Furthermore, TWA of heart rate was significantly higher in patients, who received vasopressors (*p* < 0.01) and in patients, who received albumin (*p* < 0.01; Table 3).

**Table 3:** Univariable regression model for TWA, ARV, generalized ARV and squared ARV of the intraoperative heart rate.

	Variable	Comparison	Estimate	95% CI	*p*-Value
TWA	Randomization Group	80% vs. 30% oxygen	0.12	−2.55 – 2.80	0.93
	Age		−0.09	−0.26 – 0.09	0.35
	BMI		−0.29	−0.56 – −0.01	0.04
	Sex	Male vs. Female	−0.88	−3.72 – 1.97	0.55
	Type of surgery	Laparoscopic vs. Open	−5.73	−8.44 – 3.02	< 0.01
	Coronary artery disease	Yes vs. No	−3.54	−6.60 – −0.47	0.03
	Peripheral artery disease	Yes vs. No	3.21	−0.50 – 6.92	0.09
	Stroke	Yes vs. No	3.46	−1.31 – 8.23	0.16
	Heart failure	Yes vs. No	4.38	−0.85 – 9.60	0.10
	Diabetes	Yes vs. No	3.97	0.00–5.95	0.05
	Hypertension	Yes vs. No	−1.69	−6.93 – 3.56	0.53
	Vasopressor	Yes vs. No	6.45	2.95–9.95	< 0.01
	Albumin	Yes vs. No	6.68	4.01–9.34	< 0.01
	Crystalloids		2.05	1.19–2.90	< 0.01
	TWA: pCO_2_		−0.03	−0.33 – 0.28	0.87
	TWA: pH		−36.10	−65.19 – −7.01	0.02
ARV	Randomization Group	80% vs. 30% oxygen	0.03	−0.44 – 0.50	0.90
	Age		0.07	0.04–0.10	< 0.01
	BMI		−0.05	−0.10 – −0.01	0.03
	Sex	Male vs. Female	−0.88	−1.36 – −0.40	< 0.01
	Type of surgery	Laparoscopic vs. Open	−0.17	−0.66 – 0.32	0.49
	Coronary artery disease	Yes vs. No	−0.32	−0.86 – 0.22	0.25
	Peripheral artery disease	Yes vs. No	0.12	−0.53 – 0.77	0.71
	Stroke	Yes vs. No	−0.12	−0.95 – 0.72	0.79
	Heart failure	Yes vs. No	1.12	0.21–2.02	0.02
	Diabetes	Yes vs. No	−0.47	−0.98 – 0.05	0.08
	Hypertension	Yes vs. No	0.28	−0.63 – 1.19	0.55
	Vasopressor	Yes vs. No	0.19	−0.43 – 0.82	0.55
	Albumin	Yes vs. No	0.21	−0.28 – 0.69	0.40
	Crystalloids		−0.16	−0.32 – −0.01	0.04
	TWA: pCO_2_		−0.03	−0.08 – 0.02	0.27
	TWA: pH		−4.73	−9.80 – 0.34	0.07
Generalized ARV	Randomization Group	80% vs. 30% oxygen	−0.02	−1.71 – 1.67	0.98
	Age		0.23	0.12–0.34	< 0.01
	BMI		−0.15	−0.32 – 0.03	0.10
	Sex	Male vs. Female	−3.29	−5.04 – −1.54	< 0.01
	Type of surgery	Laparoscopic vs. Open	−0.57	−2.34 – 1.19	0.53
	Coronary artery disease	Yes vs. No	−1.39	−3.34 – 0.56	0.16
	Peripheral artery disease	Yes vs. No	−0.18	−2.54 – 2.18	0.88
	Stroke	Yes vs. No	−0.76	−3.78 – 2.26	0.62
	Heart failure	Yes vs. No	3.46	0.18–6.75	0.04
	Diabetes	Yes vs. No	−1.83	−3.71 – 0.05	0.06
	Hypertension	Yes vs. No	0.89	−2.42 – 4.21	0.60
	Vasopressor	Yes vs. No	0.70	−1.57 – 2.96	0.60
	Albumin	Yes vs. No	0.64	−1.12 – 2.40	0.48
	Crystalloids		−0.52	−1.08 – 0.04	0.07
	TWA: pCO_2_		−0.17	−0.36 – 0.02	0.08
	TWA: pH		−9.14	−27.58 – 9.31	0.33
	Randomization Group	80% vs. 30% oxygen	−88.47	−311.44 – 134.549	0.44
Squared ARV	Age		28.93	14.61–43.26	< 0.01
	BMI		−19.98	−42.69 – 2.73	0.09
	Sex	Male vs. Female	−511.56	−740.60 – −282.51	< 0.01
	Type of surgery	Laparoscopic vs. Open	−191.13	−423.53 – 41.28	0.11
	Coronary artery disease	Yes vs. No	−195.73	−453.04 – 61.58	0.14
	Peripheral artery disease	Yes vs. No	40.00	−271.57 – 351.51	0.80
	Stroke	Yes vs. No	−173.05	−572.12 – 226.01	0.40
	Heart failure	Yes vs. No	398.29	−37.10 – 833.67	0.07
	Diabetes	Yes vs. No	−110.49	−360.03 – 139.04	0.39
	Hypertension	Yes vs. No	166.62	−271.02 – 604.25	0.46
	Vasopressor	Yes vs. No	80.58	−218.74 – 379.90	0.60
	Albumin	Yes vs. No	102.63	−130.04 – 335.30	0.39
	Crystalloids		−58.28	−132.21 – 15.64	<0.01
	TWA: pCO_2_		−12.76	−37.85 – 12.34	0.32
	TWA: pH		−1526.77	−3973.77 – 920.23	0.02

Estimate of slope, confidence intervals (CI) and p-values were calculated using univariable linear regression models. TWA, time-weighted average; ARV, average real variability.

In the multivariable analysis accounting for BMI, type of surgery, coronary artery disease, vasopressor use, use of albumin, amount of crystalloids and pH, all factors except for type of surgery and use of vasopressors remained significant (BMI: estimate: –0.32, CI –0.58 to −0.06, *p* = 0.02; surgical type: estimate: -5.54, CI –8.21 to −2.97, *p* < 0.01; coronary artery disease: estimate: –3.51, CI: −6.48 to −0.53, *p* = 0.02; Albumin: estimate: 3.87, CI: 0.91–6.83; *p* = 0.01; crystalloids: estimate: 1.12, CI: 0.14–2.09, *p* = 0.03), TWA: pH: estimate: –39.49, CI: −65.91 to −12.88; (Online Supplementary eTable 7).

Furthermore, no significant difference in ARV of heart rate between the 80 and 30% oxygen was found (median ARV of heart rate in the 80% oxygen group: 2.4 beats.min^−1^ [1.9; 4.3], and in the 30% oxygen group: 2.7 beats.min^−1^ [1.8; 4.3]; effect estimate: 0.03 beats.min^−^1, CI –0.44 to 0.49, *p* = 0.90; Table 3). A significantly larger ARV of the heart rate was found for older patients (*p* < 0.01), patients with lower BMI (*p* = 0.027), females (p < 0.01) as well as in patients with history of heart failure (*p* = 0.02).

In the multivariable analyses only the variables Age, Sex and Heart failure remained significant (age: estimate: 0.06, lower CL: 0.03, upper CL: 0.09, value of *p*: < 0.01; BMI: estimate: −0.04, lower CL: −0.09, upper CL: 0.01, value of p: 0.08; Sex: estimate: −0.74, lower CL: −1.21, upper CL: −0.27, value of *p*: < 0.01; History of heart failure: estimate: 0.91, lower CL: 0.04, upper CL: 1.78, value of *p*: 0.04).

The generalized ARV (*p* = 0.98) as well as the squared ARV (*p* = 0.44) of heart rate did not differ significantly between the two groups. A significantly larger generalized ARV of heart rate was found for older patients (*p* < 0.01), females (*p* < 0.01) as well as in patients with heart failure (*p* = 0.04). Only age and sex remained significant in the multivariable analysis (age: estimate: 0.22, CI 0.11 to 0.32, *p* < 0.01; sex: estimate: –2.79, CI: −4.52 to −1.11, *p* < 0.01).

A significant larger squared ARV of the heart frequency was found for older patients (*p* < 0.01) as well as female patients (*p* < 0.01). In the multivariable analyses both factors remained significant (Age: estimate: 26.72, lower CL: 12.80, upper CL: 40.64, value of p: <0.01; Sex: estimate: −479.66, lower CL: −703.65, upper CL: −255.67, value of *p*: < 0.01). Detailed results of analyses of heart rate are presented in Table 3.

## Discussion

In this exploratory analysis, we did not observe significantly higher intraoperative blood pressure values in patients, who received 80% oxygen as compared to those, who received 30% oxygen during abdominal surgery. Furthermore, we did not observe a significant effect on intraoperative heart rate as well.

In contrast to our study, several previous studies showed that hyperoxia leads to a significant increase in blood pressure and systemic vascular resistance and to a significant decrease in heart rate and cardiac output (12). These studies were relatively small including both healthy subjects and patients with coronary artery disease, heart failure, undergoing heart surgery, and with sepsis ( 12). A possible explanation for these hemodynamic effects derives from an *in vitro* study, which showed that hyperoxia leads to an increase in peripheral vasoconstriction, which simultaneously increases blood pressure and systemic vascular resistance and furthermore leads further to a reflex-like reduction in heart rate and cardiac output (13). There are several differences between our study and the fore-mentioned studies. The most important difference is that our patients had surgery under general anesthesia. It is well known that anesthetics lead to a dose dependent decrease in systolic and diastolic blood pressure, which is most likely caused by a decrease in systemic vascular resistance (22–24). Thus, it might be possible that oxygen had influenced our blood pressure, but the administration of volatile anesthetics attenuates this effect. Nonetheless, we guided anesthesia according to processed electroencephalography, which resulted in similar end tidal sevoflurane concentration in both groups (1.2% [1.1; 1.4] in the 80% oxygen group and 1.2% [1.0–1.4] in the 30% oxygen group, *p* = 0.364) (7). Therefore, it seems unlikely that intraoperative concentration of volatile anesthetics has affected our results.

A further explanation for the different results might be that our patients received supplemental oxygen during the whole surgical procedure, which was approximately 4 h in both groups. Instead, the exposure time of supplemental oxygen in the above studies ranged only between 5 and 30 min (12). The authors evaluated hemodynamic effects immediately after inducing hyperoxia but not for the long-term administration of oxygen. It might be possible that hemodynamic effects of acute hyperoxia might only last for a limited time until there is an adaptation of the peripheral vascular system to hyperoxia leading to a normalization of the vascular tension (13). We did not evaluate hemodynamic effects immediately after administration of 80% oxygen. We calculated TWA of blood pressure and ARV of blood pressure during the whole surgical procedure. Thus, it might be possible that the duration of any significant changes in hemodynamics might be too short to be found in our analysis. This further emphasizes our observations that supplemental oxygen may have no clinical meaningful effects on blood pressure during surgery.

Average-real variability (ARV) is more reliable than standard deviation to estimate variability for time series data (20). Hansen et al. (20) showed that higher ARV of systolic and diastolic blood pressure over 24 h (ARV_24_) was a predictor for mortality, cardiovascular- and cerebrovascular-events. Furthermore, Mascha et al. evaluated the ARV of MAP in over 106,000 patients, who had noncardiac surgery, and showed that generalized ARV of MAP and TWA of MAP were independently associated with 30-day mortality. In detail, they have shown that 30-day mortality increased steeply within an increase of generalized ARV to 3 mmHg.min^−1^ (21). However, when generalized ARV increases further, the association to 30-day mortality is becoming weaker (21).

Since previous studies suggested that supplemental oxygen increases systemic vascular resistance, we assumed that oxygen may thus reduce hypotensive periods leading to a lower ARV. Thus, we analyzed ARV of MAP between the groups. However, we did not observe any significant difference in ARV of blood pressure between the groups. This is reasonable since we also did not observe any significant effect on TWA of MAP as well. We further found in our multivariable analysis that age, sex, BMI, and diabetes had significantly higher generalized ARV of MAP. This is consistent with Mascha et al. (21), who found that ARV was significantly higher in a wide range of baseline comorbidities, e.g., diabetes, hypertension, vascular disease.

Several studies have shown that a low heart variability is a parameter for autonomic nervous dysfunction and is further associated with an increased risk of cardiovascular events (20). A previous study indicated that hyperoxia is an activator of the parasympathetic nervous system in dose-dependent manner (25). Thus, we expected that ARV of heart rate and TWA of heart rate will be significantly lowered by supplemental oxygen during surgery as well. However, we found no significant difference in generalized ARV of heart and in TWA of heart rate between the groups. The most reasonable explanation for our findings is that all patients received intraoperative opioids. Opioids decrease heart rate in a dose-dependent manner due to an increase in parasympathetic nerve activity (26). Furthermore, opioids blunt intraoperative hemodynamic responses to surgical interventions which further influences heart rate as well. The median dose of intraoperative fentanyl in the 80% oxygen group was 1,000 μg [800; 1,400] and 1,000 μg [800; 1,419] in the 30% oxygen group. It is very likely that the effect of opioids outweighs a possible effect of different oxygen concentrations on intraoperative TWA of heart rate and ARV of heart rate. Nevertheless, previous studies evaluated heart rate variability using ECG based RR intervals instead of the heart *per se* (20, 27). It might be possible that ECG based RR intervals might be more accurate to evaluate heart rate variability as compared to variations between heart rate *per se*. Thus, the comparison with previous studies is limited. Therefore, the clinical meaning of generalized ARV of heart rate *per se* needs to be clarified in further studies.

The results of this study must be read with some caution. First, this was an exploratory analysis of a prospective randomized controlled trial, primarily planned to investigate the effect of supplemental oxygen on postoperative maximum NT-proBNP concentration. However, the difference in TWA of MAP and ARV of MAP was observed to be only minimally different between the 80 and 30% oxygen group, thus it seems to be unlikely that a clinical relevant difference between different oxygen concentrations can be found in studies of the same kind but larger sample size.

Furthermore, we must emphasize that we did not measure systemic vascular resistance. We used esophageal doppler measurements for intraoperative hemodynamic guidance. Thus, our findings reflect possible effects of oxygen on systemic vascular resistance in an indirect manner only.

Another limitation of our study is that we compared an intraoperative FiO_2_ of 0.8 versus FiO_2_ of 0.3. In fact, oxygen partial pressures in our 30% oxygen group were mildly higher (131 mmHg [108; 160]) as compared to normal physiological partial pressure of oxygen which lies at 75–100 mmHg (7). Thus, we actually compared the effect of mild hyperoxia with relatively severe hyperoxia (314 mmHg [263; 356]) (7). Nevertheless, the administration of 0.21 FiO_2_ during surgery is very uncommon, thus our study represents the current clinical practice, in which higher concentrations are often administered due to avoid hypoxemia. Beyond that, a pronounced beneficial effect of postoperative supplemental oxygen on hemodynamics in mildly hypoxic patients, who often remain unnoticed on the surgical ward has not be studied and thus cannot be excluded.

In summary, we observed in our several analyses that intraoperative supplemental oxygen has no significant hemodynamic effects neither on TWA and ARV of MAP nor on TWA and ARV of heart rate. This is consistent with our previous studies in which we showed that postoperative NT-proBNP, Troponin T, catecholamines and copeptin concentrations were also not significantly influenced by intraoperative supplemental oxygen administration. Based on our results and recent trials it is very likely that the intraoperative used oxygen concentration is a negligible factor regarding influencing clinical meaningful events.

## Data availability statement

The raw data supporting the conclusions of this article will be made available by the authors, without undue reservation.

## Ethics statement

The studies involving human participants were reviewed and approved by Ethics committee of the Medical University of Vienna. The patients/participants provided their written informed consent to participate in this study.

## Author contributions

CR: conceptualization, material preparation, data collection, data analysis, writing the first draft and final manuscript. EF and BK: conceptualization, material preparation, writing the first draft and final manuscript. AT, NA, and MS: material preparation, data collection, revision of the manuscript AK: material preparation, data interpretation, writing the first draft and final manuscript. MF: conceptualizaiton, data management, data analysis, revision of the manuscript AG: conceptualizaiton, data analysis plan and data analysis, revision of the manuscript. All authors contributed to the article and approved the submitted version.

## Conflict of interest

The authors declare that the research was conducted in the absence of any commercial or financial relationships that could be construed as a potential conflict of interest.

## Publisher’s note

All claims expressed in this article are solely those of the authors and do not necessarily represent those of their affiliated organizations, or those of the publisher, the editors and the reviewers. Any product that may be evaluated in this article, or claim that may be made by its manufacturer, is not guaranteed or endorsed by the publisher.
