# Supplementary material for: Hemodynamic effects of intraoperative 30% versus 80% oxygen concentrations: an exploratory analysis
Source: Front Med (Lausanne). 2023 May 30;10:1200223. doi: 10.3389/fmed.2023.1200223 (PMC10265637; doi:10.3389/fmed.2023.1200223)
Supplement: Supplementary file 1 [file Table_1.DOCX]

**Supplementary Online Content**

**eTable 1:** Descriptive statistics of TWA of arterial pCO2, TWA of arterial pH, amount of phenylephrine and noradrenaline, amount of Albumin and crystalloids.

**eTable 2:** Multivariable regression model for TWA, ARV, generalized ARV and squared ARV of the intraoperative mean arterial pressure.

**eTable 3:** Univariable regression model for TWA, ARV, generalized ARV and squared ARV of the intraoperative systolic arterial blood pressure.

**eTable 4:** Multivariable regression model for TWA, ARV, generalized ARV and squared ARV of the intraoperative systolic arterial pressure.

**eTable 5:** Univariable regression model for TWA, ARV, generalized ARV and squared ARV of the intraoperative diastolic arterial blood pressure.

**eTable 6:** Multivariable regression model for TWA, ARV, generalized ARV and squared ARV of the intraoperative diastolic arterial blood pressure.

**eTable 7:** Multivariable regression model for TWA, ARV, generalized ARV and squared ARV of heart rate.

| **Variable** | **Group** | | **Mean** | | **SD** | | **Min** | | **Q1** | | **Median** | | **Q3** | | **Max** | | **n** | |
| --- | --- | --- | --- | --- | --- | --- | --- | --- | --- | --- | --- | --- | --- | --- | --- | --- | --- | --- |
| TWA: pCO_2_ | 30% | 42.2 | | 3.91 | | 31.7 | | 39.68 | | 42 | | 44.35 | | 59.37 | | 130 | |  |
|  | 80% | 43.96 | | 4.82 | | 34.06 | | 40.82 | | 43.44 | | 45.95 | | 64.13 | | 127 | |  |
| TWA: pH | 30% | 7.37 | | 0.04 | | 7.22 | | 7.35 | | 7.38 | | 7.4 | | 7.46 | | 130 | |  |
|  | 80% | 7.36 | | 0.05 | | 7.14 | | 7.33 | | 7.36 | | 7.38 | | 7.5 | | 127 | |  |
| Phenylephrine, mg | 30% | 0.35 | | 0.31 | | 0.04 | | 0.12 | | 0.3 | | 0.5 | | 1.96 | | 96 | |  |
|  | 80% | 0.54 | | 1.1 | | 0.04 | | 0.18 | | 0.3 | | 0.57 | | 10 | | 95 | |  |
| Noradrenaline, mg | 30% | 0.85 | | 0.88 | | 0.03 | | 0.2 | | 0.6 | | 1.1 | | 3.8 | | 63 | |  |
|  | 80% | 0.96 | | 1 | | 0.1 | | 0.3 | | 0.6 | | 1.1 | | 4.3 | | 58 | |  |
| Albumin, mL | 30% | 265.85 | | 145.96 | | 100 | | 200 | | 200 | | 300 | | 700 | | 41 | |  |
|  | 80% | 208.8 | | 107.55 | | 100 | | 150 | | 200 | | 200 | | 600 | | 51 | |  |
| Crystalloids, L | 30% | 2.58 | | 1.44 | | 0 | | 1.61 | | 2.37 | | 3.32 | | 8.68 | | 130 | |  |
|  | 80% | 2.54 | | 1.57 | | 0.43 | | 1.38 | | 2.175 | | 3.30 | | 10.5 | | 128 | |  |
| SVR, dynsec.cm^-5^ | 30% | 1637 | | 730 | | 617 | | 1153 | | 1479 | | 1915 | | 4787 | | 130 | |  |
|  | 80% | 1422 | | 532 | | 670 | | 1053 | | 1370 | | 1915 | | 3366 | | 128 | |  |

**eTable 1:** Descriptive statistics of TWA of arterial pCO2, TWA of arterial pH, amount of phenylephrine and noradrenaline, amount of Albumin, crystalloids and SVR.

**eTable 2:** Multivariable regression model for TWA, ARV, generalized ARV and squared ARV of the intraoperative mean arterial pressure.

|  | | | | | | | |
| --- | --- | --- | --- | --- | --- | --- | --- |
|  | **Variable** | **Comaprison** | **Estimate** | **lower_CL** | **upper_CL** | **p_value** |  |
| TWA | Type of Surgery | Laparoscopic vs. Open | 1.697 | -0.132 | 3.526 | 0.0701 |  |
|  | Vasopressor | Yes vs. No | -3.784 | -5.992 | -1.576 | 0.0009 |  |
|  | Albumin | Yes vs. No | 0.050 | -1.870 | 1.969 | 0.9595 |  |
|  | Crystalloids |  | -0.580 | -1.204 | 0.044 | 0.0697 |  |
|  | TWA: pH |  | 24.231 | 7.034 | 41.428 | 0.0062 |  |
| ARV | Age |  | 0.024 | 0.005 | 0.043 | 0.0126 |  |
|  | Crystalloids |  | -0.145 | -0.240 | -0.050 | 0.0031 |  |
| Generalized ARV | Age |  | 0.079 | 0.032 | 0.126 | 0.0012 |  |
|  | BMI |  | -0.087 | -0.159 | -0.015 | 0.0193 |  |
|  | Sex | Male vs. Female | -0.669 | -1.402 | 0.064 | 0.0748 |  |
|  | Diabetes | Yes vs. No | -0.319 | -1.117 | 0.479 | 0.4338 |  |
|  | Crystalloids |  | -0.260 | -0.492 | -0.028 | 0.0290 |  |
| Squared ARV | BMI |  | -6.335 | -14.324 | 1.654 | 0.1214 |  |
|  | Crystalloids |  | -48.840 | -74.785 | -22.895 | 0.0003 |  |

**eTable 3:** Univariable regression model for TWA, ARV, generalized ARV and squared ARV of the intraoperative systolic arterial blood pressure.

| **Value** | **Variable** | **Comaprison** | **Estimate** | **95% CI** | **p_value** |
| --- | --- | --- | --- | --- | --- |
| TWA | Randomization | 80% vs. 30% | -1.30 | -4.15 – 1.55 | 0.37 |
|  | Age |  | 0.48 | 0.30 – 0.66 | <0.01 |
|  | BMI |  | -0.22 | -0.52 – 0.07 | 0.13 |
|  | Sex | Male vs. Female | 1.60 | -1.43 – 4.62 | 0.30 |
|  | Type of Surgery | Laparoscopic vs. Open | 2.82 | -0.15 – 5.79 | 0.07 |
|  | Coronary Artery Disease | Yes vs. No | 2.62 | -0.68 – 5.92 | 0.12 |
|  | Peripheral Artery Disease | Yes vs. No | 0.50 | -3.47 – 4.47 | 0.81 |
|  | Stroke | Yes vs. No | -2.49 | -7.58 – 2.60 | 0.34 |
|  | Heart failure | Yes vs. No | 4.38 | -1.18 – 9.94 | 0.12 |
|  | Diabetes | Yes vs. No | -2.25 | -5.43 – 0.92 | 0.165 |
|  | Hypertension | Yes vs. No | 7.52 | 2.01 – 13.03 | <0.01 |
|  | Vasopressor | Yes vs. No | -7.53 | -11.27 – -3.79 | <0.01 |
|  | Albumin | Yes vs. No | -2.56 | -5.51 – 0.40 | 0.09 |
|  | Crystalloids |  | -2.30 | -3.20 – -1.39 | <0.01 |
|  | TWA: pCO_2_ |  | 0.27 | -0.05 – 0.59 | 0.10 |
|  | TWA: pH |  | -5.54 | -36.78 – 25.69 | 0.73 |
| ARV | Randomization | 80% vs. 30% | -0.24 | -0.65 – 0.18 | 0.26 |
|  | Age |  | 0.03 | -0.01 – 0.05 | 0.18 |
|  | BMI |  | -0.04 | -0.08 – 0.01 | 0.08 |
|  | Sex | Male vs. Female | -0.16 | -0.60 – 0.27 | 0.46 |
|  | Type of Surgery | Laparoscopic vs. Open | 0.35 | -0.08 – 0.78 | 0.11 |
|  | Coronary Artery Disease | Yes vs. No | -0.29 | -0.77 – 0.19 | 0.23 |
|  | Peripheral Artery Disease | Yes vs. No | 0.51 | -0.06 – 1.08 | 0.08 |
|  | Stroke | Yes vs. No | -0.32 | -1.06 – 0.41 | 0.39 |
|  | Heart failure | Yes vs. No | 0.02 | -0.78 – 0.83 | 0.96 |
|  | Diabetes | Yes vs. No | -0.31 | -0.77 – 0.15 | 0.19 |
|  | Hypertension | Yes vs. No | -0.96 | -1.76 –-0.16 | 0.02 |
|  | Vasopressor | Yes vs. No | -0.26 | -0.81 – 0.30 | 0.37 |
|  | Albumin | Yes vs. No | -0.17 | -0.60 – 0.26 | 0.44 |
|  | Crystalloids |  | -0.17 | -0.30 – -0.03 | 0.02 |
|  | TWA: pCO_2_ |  | 0.01 | -0.04 – 0.06 | 0.66 |
|  | TWA: pH |  | -3.30 | -7.78 – 1.18 | 0.15 |
| Generalzed ARV | Randomization | 80% vs. 30% | -0.39 | -1.50 – 0.73 | 0.50 |
|  | Age |  | 0.11 | 0.034 – 0.18 | <0.01 |
|  | BMI |  | -0.16 | -0.276 | <0.01 |
|  | Sex | Male vs. Female | -1.07 | -2.24 – 0.11 | 0.08 |
|  | Type of surgery | Laparoscopic vs. Open | 0.04 | -1.13 – 1.21 | 0.95 |
|  | Coronary Artery Disease | Yes vs. No | -0.88 | -2.17 – 0.42 | 0.19 |
|  | Peripheral Artery Artery Disease | Yes vs. No | 0.32 | -1.23 – 1.88 | 0.69 |
|  | Stroke | Yes vs. No | -1.24 | -3.23 – 0.75 | 0.22 |
|  | Heart failure | Yes vs. No | -0.23 | -2.41 – 1.20 | 0.84 |
|  | Diabetes | Yes vs. No | -1.16 | -2.40 – 0.08 | 0.07 |
|  | Hypertension | Yes vs. No | -0.76 | -2.95 – 1.42 | 0.49 |
|  | Vasopressor | Yes vs. No | 1.42 | -0.08 – 2.92 | 0.06 |
|  | Albmin | Yes vs. No | -0.02 | -1.18 – 1.15 | 0.98 |
|  | Crystalloids |  | -0.44 | -0.81 – -0.07 | 0.02 |
|  | TWA: pCO_2_ |  | -0.05 | -0.18 – 0.07 | 0.42 |
|  | TWA: pH |  | -6.37 | -18.56 – 5.83 | 0.31 |
| Squared ARV | Randomization | 80% vs. 30% | -148.49 | -376.28 – 79.31 | 0.20 |
|  | Age |  | 8.32 | -6.73 – 23.6 | 0.28 |
|  | BMI |  | -12.22 | -35.59 – 11.14 | 0.31 |
|  | Sex | Male vs. Female | -52.10 | -294.86 – 190.67 | 0.67 |
|  | Type of Surgery | Laparoscopic vs. Open | 230.22 | -7.61 – 468.06 | 0.06 |
|  | Coronary Artery Disease | Yes vs. No | -113.69 | -378.92 – 151.55 | 0.40 |
|  | Peripheral Artery Disease | Yes vs. No | 54.04 | -264.34 – 372.42 | 0.74 |
|  | Stroke | Yes vs. No | 88.68 | -319.55 – 496.91 | 0.67 |
|  | Heart failure | Yes vs. No | -191.63 | -638.69 – 255.43 | 0.40 |
|  | Diabetes | Yes vs. No | -89.96 | -345.24 – 165.32 | 0.49 |
|  | Hypertension | Yes vs. No | -144.55 | -591.87 – 302.78 | 0.53 |
|  | Vasopressor | Yes vs. No | 108.09 | -200.63 – 416.81 | 0.49 |
|  | Albumin | Yes vs. No | -105.80 | -343.77 – 132.17 | 0.38 |
|  | Crystalloids |  | -98.30 | -173.37 – -23.21 | 0.01 |
|  | TWA: pCO_2_ |  | -11.62 | -37.23 – 13.99 | 0.37 |
|  | TWA: pH |  | -1877.16 | -4370.41 – 616.04 | 0.14 |

**eTable 4:** Multivariable regression model for TWA, ARV, generalized ARV and squared ARV of the intraoperative systolic arterial pressure.

| **Value** | **Variable** | **Comaprison** | **Estimate** | **95% CI** | **P value** |
| --- | --- | --- | --- | --- | --- |
| TWA | Age |  | 0.41 | 0.24 – 0.58 | <0.01 |
|  | Hypertension | Yes vs. No | 5.74 | 0.69 – 10.79 | 0.03 |
|  | Vasopressor | Yes vs. No | -5.49 | -9.05 – -1.94 | <0.01 |
|  | Crystalloids |  | -1.77 | -2.65 – -0.89 | <0.01 |
| ARV | Hypertension | Yes vs. No | -0.93 | -1.72 – -0.14 | 0.02 |
|  | Crystalloids |  | -0.16 | -0.30 – -0.03 | 0.02 |
| Generalzed ARV | Age |  | 0.09 | 0.02 – 0.16 | 0.02 |
|  | BMI |  | -0.13 | -0.24 – --0.02 | 0.03 |
|  | Crystalloids |  | -0.34 | -0.70 – 0.03 | 0.07 |

**eTable 5:** Univariable regression model for TWA, ARV, generalized ARV and squared ARV of the intraoperative diastolic arterial blood pressure.

| **Value** | **Variable** | **Comaprison** | **Estimate** | | **95% CI** | | **P value** | |
| --- | --- | --- | --- | --- | --- | --- | --- | --- |
| TWA | Randomization | 80% vs. 30% | -0.13 | -1.78 – 1.53 | | 0.88 | |  |
|  | Age |  | -0.10 | -0.21 – 0.01 | | 0.06 | |  |
|  | BMI |  | 0.13 | -0.04 – 0.30 | | 0.12 | |  |
|  | Sex | M vs. F | 2.78 | 1.05 – 4.50 | | <0.01 | |  |
|  | Type of Surgery | Laparoscopic vs. open | 3.03 | 1.33 – 4.72 | | <0.01 | |  |
|  | Coronary Artery Disease | Yes vs. No | -0.78 | -2.70 – 1.14 | | 0.43 | |  |
|  | Peripheral Artery Disease | Yes vs. No | -0.73 | -3.03 – 1.58 | | 0.54 | |  |
|  | Stroke | Yes vs. No | 0.71 | -2.25 – 3.67 | | 0.64 | |  |
|  | Heart failure | Yes vs. No | 0.57 | -2.67 – 3.82 | | 0.73 | |  |
|  | Diabetes | Yes vs. No | -0.50 | -2.35 – 1.35 | | 0.60 | |  |
|  | Hypertension | Yes vs. No | 0.85 | -2.39 – 4-09 | | 0.61 | |  |
|  | Vasopressor | Yes vs. No | -3.80 | -5.99 – -1.61 | | <0.01 | |  |
|  | Albumin | Yes vs. No | -1.56 | -3.28 – 0.15 | | 0.08 | |  |
|  | Crystalloids |  | -0.31 | -0.86 – 0.24 | | 0.27 | |  |
|  | TWA: pCO_2_ |  | -0.08 | -0.26 – 0.11 | | 0.41 | |  |
|  | TWA: pH |  | 32.48 | 14.79 – 50.18 | | <0.01 | |  |
| ARV | Randomization | 80% vs. 30% | 0.04 | -0.25 – 0.34 | | 0.79 | |  |
|  | Age |  | 0.02 | 0.01 – 0.04 | | 0.02 | |  |
|  | BMI |  | -0.01 | -0.04 – 0.02 | | 0.53 | |  |
|  | Sex | Male vs. Female | -0.12 | -0.43 – 0.20 | | 0.47 | |  |
|  | Type of Surgery | Laparoscopic vs. Open | 0.36 | 0.07 – 0.67 | | 0.02 | |  |
|  | Coronary Artery Disease | Yes vs. No | -0.20 | -0.55 – 0.14 | | 0.24 | |  |
|  | Peripheral Artery Disease | Yes vs. No | -0.11 | -0.52 – 0.30 | | 0.60 | |  |
|  | Stroke | Yes vs. No | -0.18 | -0.70 – 0.36 | | 0.52 | |  |
|  | Heart failure | Yes vs. No | -0.03 | -0.61 – 0.54 | | 0.91 | |  |
|  | Diabetes | Yes vs. No | -0.30 | -0.63 – 0.03 | | 0.07 | |  |
|  | Hypertension | Yes vs. No | -0.14 | -0.72 – 0.43 | | 0.64 | |  |
|  | Vasopressor | Yes vs. No | -0.19 | -0.60 – 0.20 | | 0.33 | |  |
|  | Albumin | Yes vs. No | -0.09 | -0.39 – 0.22 | | 0.59 | |  |
|  | Crystalloids |  | -0.17 | -0.26 – -0.07 | | <0.01 | |  |
|  | TWA: pCO2 |  | 0.01 | -0.03 – 0.04 | | 0.77 | |  |
|  | TWA: pH |  | -0.61 | -3.84 – 2.62 | | 0.71 | |  |
| Generalized ARV | Randomization | 80% vs. 30% | 0.02 | -0.63 – 0.66 | | 0.96 | |  |
|  | Age |  | 0.06 | 0.02 – 0.11 | | <0.01 | |  |
|  | BMI |  | -0.06 | -0.13 – 0.01 | | 0.07 | |  |
|  | Sex | Male vs. Female | -0.52 | -1.21 – 0.16 | | 0.14 | |  |
|  | Type of Surgery | Laparoscopic vs. Open | 0.81 | 0.14 – 0.49 | | 0.02 | |  |
|  | Coronary Artery Disease | Yes vs. No | -0.46 | -1.21 – 0.29 | | 0.23 | |  |
|  | Peripheral Artery Disease | Yes vs. No | -0.26 | -1.16 – 0.64 | | 0.58 | |  |
|  | Stroke | Yes vs. No | -0.50 | -1.66 – 0.66 | | 0.40 | |  |
|  | Heart failure | Yes vs. No | -0.18 | -1.45 – 1.09 | | 0.78 | |  |
|  | Diabetes | Yes vs. No | -0.79 | -1.51 – -0.07 | | 0.03 | |  |
|  | Hypertension | Yes vs. No | -0.38 | -1.65 – 0.89 | | 0.56 | |  |
|  | Vasopressor | Yes vs. No | -0.03 | -0.90 – 0.85 | | 0.95 | |  |
|  | Albumin | Yes vs. No | -0.54 | -1.22 – 0.13 | | 0.12 | |  |
|  | Crystalloids |  | -0.43 | -0.64 – -0.22 | | <0.01 | |  |
|  | TWA: pCO_2_ |  | -0.04 | -0.11 – 0.04 | | 0.32 | |  |
|  | TWA: pH |  | -0.94 | -8.05 – 6.17 | | 0.80 | |  |
| Squared ARV | Randomization | 80% vs. 30% | -2.64 | -70.15 – 64.87 | | 0.94 | |  |
|  | Age |  | 1.58 | -2.87 – 6.03 | | 0.49 | |  |
|  | BMI |  | -3.60 | -10.51 – 3.30 | | 0.31 | |  |
|  | Sex | Male vs. Female | 4.98 | -66.76 – 76.72 | | 0.89 | |  |
|  | Type of Surgery | Laparoscopic vs. Open | 127.18 | 58.17 – 196.20 | | <0.01 | |  |
|  | Coronary Artery Disease | Yes vs. No | -10.62 | -89.07 – 67-84 | | 0.79 | |  |
|  | Peripheral Artery Artery Disease | Yes vs. No | 0.93 | -93.15 – 95.01 | | 0.99 | |  |
|  | Stroke | Yes vs. No | -29.20 | -149.79 – 91.39 | | 0.64 | |  |
|  | Heart failure | Yes vs. No | -48.56 | -180.69 – 83.56 | | 0.47 | |  |
|  | Diabetes | Yes vs. No | -40.33 | -115.65 – 35.00 | | 0.30 | |  |
|  | Hypertension | Yes vs. No | -31.97 | -164.17 – 100.23 | | 0.64 | |  |
|  | Vasopressor | Yes vs. No | -84.71 | -175.40 – 5.99 | | 0.07 | |  |
|  | Albumin | Yes vs. No | -69.61 | -139.50 – 0.28 | | 0.05 | |  |
|  | Crystalloids |  | -39.83 | -61.76 – -17.90 | | <0.01 | |  |
|  | TWA: pCO_2_ |  | -1.76 | -9.34 – 5.81 | | 0.65 | |  |
|  | TWA: pH |  | -146.18 | -885.68 – 593.32 | | 0.70 | |  |

**eTable 6:** Univariable regression model for TWA, ARV, generalized ARV and squared ARV of the intraoperative diastolic arterial blood pressure.

| **Value** | **Variable** | **Comaprison** | **Estimate** | **95% CI** | | **P value** | |
| --- | --- | --- | --- | --- | --- | --- | --- |
| TWA | Sex | Male vs. Female | 2.54 | 0.91 – 4.18 | <0.01 | |  |
|  | Type of Surgery | Laparoscopic vs. Open | 2.47 | 0.83 – 4.12 | <0.01 | |  |
|  | Vasopressor | Yes vs. No | -2.96 | -5.079 – -0.84 | <0.01 | |  |
|  | TWA: pH |  | 32.21 | 15.40 – 49.01 | <0.01 | |  |
| ARV | Age |  | 0.02 | 0.01 – 0.04 | 0.03 | |  |
|  | Type of Surgery | Laparoscopic vs. Open | 0.25 | -0.08 – 0.57 | 0.14 | |  |
|  | Crystalloids |  | -0.12 | -0.23 – -0.20 | 0.02 | |  |
| Generalized ARV | Age |  | 0.05 | 0.01 – 0.10 | 0.01 | |  |
|  | Type of Surgery | Laparoscopic vs. Open | 0.49 | -0.22 – 1.20 | 0.18 | |  |
|  | Diabetes | Yes vs. No | -0.45 | -1.17 – 0.27 | 0.22 | |  |
|  | Crystalloids |  | -0.32 | -0.55 – -0.10 | <0.01 | |  |
| Squared ARV | Type of Surgery | Laparoscopic vs. Open | 93.16 | 19.34 – 166.97 | 0.01 | |  |
|  | Crystalloids |  | -28.70 | -52.13 – -5.26 | 0.02 | |  |

**eTable 7:** Multivariable regression model for TWA, ARV, generalized ARV and squared ARV of heart rate.

| **Value** | | **Variable** | | **Comaprison** | | **Estimate** | | **95% CI** | | **P value** | |
| --- | --- | --- | --- | --- | --- | --- | --- | --- | --- | --- | --- |
| TWA | BMI | |  | | -0.36 | | -0.62 – -0.11 | | 0.01 | |  |
|  | Type of Surgery | | Laparoscopic vs. Open | | -2.41 | | -5.24 – 0.41 | | 0.10 | |  |
|  | Coronary Artery Disease | | Yes vs. No | | -3.67 | | -6.49 – -0.84 | | 0.01 | |  |
|  | Vasopressor | | Yes vs. No | | 3.43 | | -0.01 – 6.86 | | 0.05 | |  |
|  | Albumin | | Yes vs. No | | 3.87 | | 0.91 – 6.83 | | 0.01 | |  |
|  | Crystalloids | |  | | 1.12 | | 0.14 – 2.09 | | 0.03 | |  |
|  | TWA: pH | |  | | -39.40 | | -65.91 – -12.88 | | 0.01 | |  |
| ARV | Age | |  | | 0.06 | | 0.03 – 0.09 | | <0.01 | |  |
|  | BMI | |  | | -0.04 | | -0.08 – 0.01 | | 0.11 | |  |
|  | Sex | | Male vs. Female | | -0.72 | | -1.20 – 0.25 | | <0.01 | |  |
|  | Heart failure | | Yes vs. No | | 0.87 | | 0.00 – 1.74 | | 0.05 | |  |
|  | Crystalloids | |  | | -0.08 | | -0.23 – 0.06 | | 0.27 | |  |
| Generalized ARV | Age | |  | | 0.22 | | 0.11 – 0.32 | | <0.01 | |  |
|  | Sex | | Male vs. Female | | -2.79 | | -4.52 – -1.07 | | <0.01 | |  |
|  | Heart failure | | Yes vs. No | | 2.69 | | -0.48 – 5.87 | | 0.10 | |  |
| Squared ARV | Age | |  | | 26.72 | | 12.80 – 40.64 | | <0.01 | |  |
|  | Sex | | Male vs. Female | | -479.66 | | -703.65 – -255.66 | | <0.01 | |  |
